# Finger Unit Design for Hybrid-Driven Dexterous Hands

**DOI:** 10.3390/biomimetics11010035

**Published:** 2026-01-04

**Authors:** Chong Deng, Wenhao Lu, Yizhou Qian, Yongjian Liu, Meng Ning, Ziheng Zhan

**Affiliations:** 1 School of Intelligent Manufacturing, Jiangnan University, Wuxi 214122, China; 6230805042@stu.jiangnan.edu.cn (C.D.); 6240805068@stu.jiangnan.edu.cn (W.L.); 6240809034@stu.jiangnan.edu.cn (Y.L.); 2 School of Mechanical, Materials and Manufacturing Engineering, University of Nottingham Ningbo China, Ningbo 315000, China; ssyyq9@nottingham.edu.cn; 3 Jiangsu Key Laboratory of Advanced Food Manufacturing Equipment and Technology, Wuxi 214122, China

**Keywords:** dexterous single-fingered hand, link-driven, tendon-driven, pendulum-measuring, anthropomorphic

## Abstract

Dexterous hands are the core end-effectors of humanoid robots, and their design is a key research focus in this field. With multiple independent finger units, the units’ dexterity directly determines the hand’s operational performance, yet achieving three-degree-of-freedom (3-DOF) anthropomorphic motion remains a key design challenge. To address this, this paper proposes a hybrid-driven index finger unit: combining linkage and tendon–cable drive advantages to realize 3-DOF anthropomorphic motion, and adopting independent drive/transmission modules to simplify manufacturing and boost parameter optimization flexibility. Validated via motion dynamics, DOF, and operational force assessments, this design offers key unit tech for dexterous hand development and serves as a reference for optimizing multi-DOF anthropomorphic finger designs.

## 1. Introduction

In recent years, dexterous hands have become core effectors for humanoid robots, with their design and development a key research focus in the field [1]. However, dexterous hand development faces challenges—especially in industrial and hazardous environments requiring both high flexibility and strong manipulative force—and synergistic optimization of these two properties remains a major bottleneck [2,3,4,5]. To tackle this, researchers have developed various dexterous manipulator configurations, whose drive mechanisms fall into three types: direct motor drive, tendon–cable drive, and link-driven mechanisms.

Directly motor-driven dexterous hand fingers achieve joint actuation via gear transmission or pulley mechanisms. Represented by LEAP HAND [6] and John’s MPL v2.0 [7], these designs have high joint actuation efficiency. However, individual finger size and performance rely on drive unit parameters: meeting high load requirements needs larger, heavier drive units, which challenges structural integration, and increased inertia also harms motion control performance.

The core feature of tendon-driven dexterous fingers lies in simulating human finger motion principles, offering anthropomorphic motion advantages. Representative examples include Cao Shengke’s team’s dexterous hand [8], NASA’s RoboNaut [9], and ShadowHand [10]. However, technical bottlenecks persist: achieving multi-degree-of-freedom motion in a single finger requires complex tendon routing and numerous actuators. To optimize weight and size, actuators are often embedded in robotic arms rather than hands, limiting interoperability during multi-robot coordination and replacement.

Dexterous finger drive schemes based on linkage mechanisms are a recent core research focus, represented by “Dexterous Heart and Hand” [11] and ILDA’s dexterous hand [12]; both use multiple four-bar linkages with three linear actuators for single-finger actuation, enabling 3-DOF motion control while maintaining stable high-force output [13,14,15]. Linkage mechanisms excel in power transmission efficiency and easy manufacturing and maintenance, making them an industry focus. However, rigid linkages’ inherent traits cause poor shock absorption in fingers—they fail to absorb impact energy in unexpected collisions, raising mechanical damage risk and reducing dexterous hand reliability.

**Figure 1:**
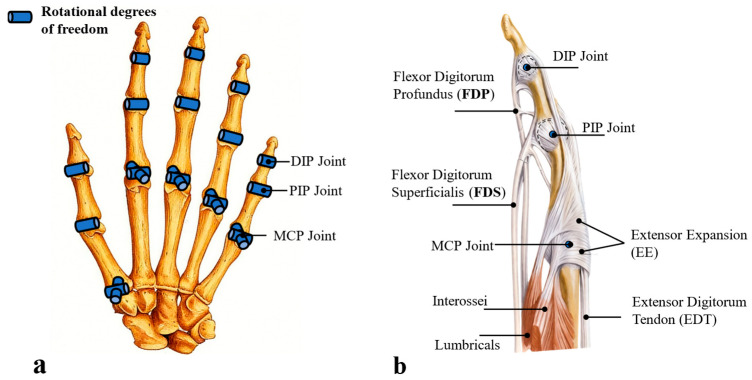
Hand mechanism diagram. (**a**) represents the overall degree of freedom distribution as defined, (**b**) depicts the anatomical diagram of a single finger.

To boost the bionic performance of dexterous hand design, we first analyze human hand anatomy: the human hand inherently has 27-DoFs, which scholars simplify to 6–20-DOFs. We thus adopt a scheme of 3-DoFs per finger and 15-DoFs in total (Figure 1a).

Single-finger muscular anatomy (Figure 1b): Metacarpophalangeal (MCP) joint flexion/abduction, driven by interossei and lumbricals, enables independent movement. Distal/proximal interphalangeal (DIP/PIP) joints are controlled by extrinsic muscles (FDP, FDS): FDS splits at PIP, wraps FDP, and inserts on the middle phalanx, so its contraction synchronizes FDP to form inseparable coupled DIP/PIP motion. Finger extension relies on coordinated extensor expansion and extensor digitorum tendon.

Based on the above analysis, we conducted a design study using the index finger unit as the research subject. The design contributions to single-finger units in this paper are as follows:

The index finger unit uses a differentiated drive system: the DIP joint and PIP joint are linked by a mechanism and precisely controlled by linear actuators; the MCP joint uses a tendon-rope drive for flexion–extension, with lateral swing enabled by a crank–rocker structure. The linkage mechanism ensures high operating force for heavy loads, while the tendon-rope drive maintains finger flexibility and improves impact resistance, balancing operational rigidity and motion safety.

To enable dimensional adjustments, structural optimization, and ease of manufacturing/debugging, this solution uses a modular design that separates the knuckle and drive structures. The drive module integrates two rotary motors and one linear motor to efficiently use limited installation space, with its power output interface designed for easy installation to lower subsequent assembly and maintenance costs. Finally, this paper analyzes and evaluates the performance of the index finger unit.

To visualize the core characteristics of different dexterous hand designs, Table 1 comprehensively compares mainstream technical schemes. Each scheme offers distinct merits requiring context-specific tradeoffs: compared to cable-driven solutions, this design slightly reduces degrees of freedom but eliminates the arm motor housing—enabling significant structural miniaturization and enhanced driving force. Against link-driven designs, the structurally analogous ILDA Hand is used for direct comparison; while its fingertip force is marginally lower, the proposed design offers superior impact resistance. Compared to direct-drive approaches, the finger dimensions are slightly larger with reduced degrees of freedom, yet the fingertip force remains sufficient for practical use. Overall, the design delivers stable performance, providing valuable insights for future hybrid-driven dexterous hand research.

## 2. Structural Design of the Finger Unit

The dexterous hand consists of multiple independent finger units, whose performance directly affects the entire hand’s functionality. Thus, this study focuses on the index finger unit’s design and analysis. To achieve high anthropomorphism in three aspects—external dimensions, joint DOF configuration, and joint motion range—it draws on NASA’s human hand biomechanics and kinematics findings [16]. Based on this, structural design metrics for the index finger unit were established, with comparisons between the designed index finger and an adult human index finger shown in Table 2. Subsequently, the index finger’s structural design and performance analysis under hybrid drive modes were completed.

**Figure 2:**
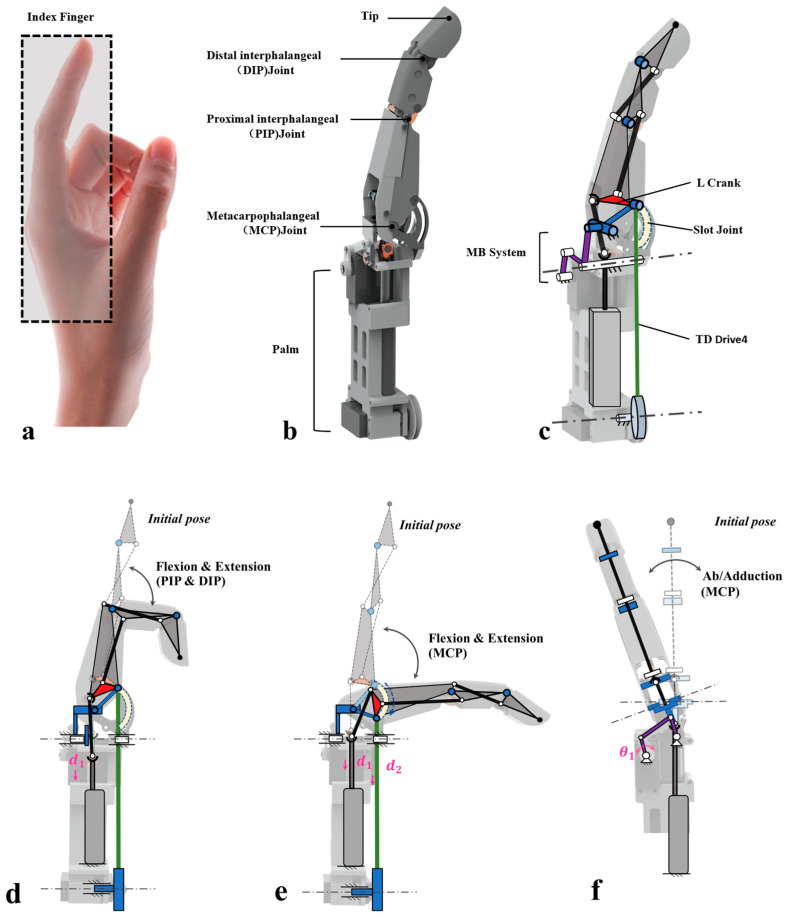
Proposed index finger structure based on hybrid drive modes, elucidates its overall configuration characteristics and the working mechanism of three-degree-of-freedom motion. Figure (**a**) shows a real human hand. (**b**) shows the overall configuration of the structural design. (**c**) depicts the motion mechanism of the index finger. (**d**) illustrates the underactuated flexion–extension motion of the DIP and PIP joints under link-driven actuation. (**e**) shows the flexion–extension motion of the MCP joint under tendon-rope actuation. (**f**) demonstrates the abduction–adduction motion of the MCP joint.

The hybrid drive design proposed in this study integrates the widely used link-driven mechanism in dexterous hands with tendon-driven mechanisms to achieve three-degree-of-freedom (3-DOF) motion for a single finger. In terms of performance advantages, this hybrid drive design retains the superior load-bearing capacity of the linkage mechanism while also featuring its robustness, ease of fabrication, and maintenance convenience. It inherits the compliance and impact resistance of the tendon–cable drive mechanism, effectively adapting to the demands of complex operational scenarios. However, the core challenge addressed in this study is achieving effective integration of both drive mechanisms within the limited structural space of a single finger while ensuring sufficient effective motion space for the finger and fully leveraging the performance characteristics of each mechanism. To overcome this technical difficulty, the specific design of the index finger single-finger structure was completed based on the schematic diagram in Figure 2.

The overall configuration of this single-finger index finger structure is shown in Figure 2a–c, with its kinematic design detailed in Figure 2d–f. This single-finger structure replicates the joint configuration of the human finger, incorporating three core joints: the distal interphalangeal joint (DIP), proximal interphalangeal joint (PIP), and metacarpophalangeal joint (MCP). The drive mechanism is integrated within the palm to optimize structural compactness. Based on kinematic studies of human fingers, the DIP and PIP joints exhibit coupled underactuated characteristics [17,18]. The mechanism employs a linkage system for actuation, with PIP joint motion maintaining relative independence from the MCP joint.

To achieve this biomimetic motion function, differentiated drive solutions must be matched to the movement requirements of each joint: First, a linear drive motor transmits power through a double ball-and-socket linkage to drive the linkage mechanism and L-shaped crank, enabling coupled motion of the PIP and DIP joints. Second, a rotary motor utilizes the cable-pulley transmission of the rudder to drive the tendon-rope (represented by the green line), enabling the MCP joint to perform flexion. Simultaneously, the MCP joint’s return motion is achieved through an internal torsion spring, whose configuration design is derived in detail in Section 2.2.1.

Additionally, the lateral swing motion required for the MCP joint is achieved through the coordinated action of a rotary motor and a four-bar linkage mechanism, whose design principles are detailed in Section 2.2.2. Thus, the entire mechanism comprises single-degree-of-freedom PIP motion and two-degree-of-freedom MCP motion generated by the hybrid drive. The linear displacement and angular displacement at the two joints ($d_{1}$, $d_{2}$, $\theta_{1}$) form the 3DOF motion of the index finger mechanism, producing high force output. The motion of each degree of freedom is illustrated in Figure 2d–f.

During linear motion ($d_{3}$) of the linear motor, the double-ball-jointed link’s end connects to the left node of the L-shaped crank via a pivot joint. Motor downward movement drives the L-shaped crank to rotate around its right node, actuating the finger root four-bar linkage to achieve independent flexion and extension of the proximal interphalangeal joint (PIP). Simultaneously, the middle phalanx’s additional four-bar linkage structure, through motion coupling, coordinates PIP and distal interphalangeal joint (DIP) movements, achieving underactuated control of both joints. Metacarpophalangeal (MCP) joint flexion/extension is achieved via tendon-rope linear motion ($d_{2}$): the tendon-rope drives the right node of the L-shaped crank to slide along a predetermined trajectory within the slide groove. MCP and PIP movements are decoupled, ensuring mutual interference-free operation. When the lateral swing motor operates, it outputs $\theta_{1}$ angular rotational motion through the crank, driving the rocker arm to swing and enabling adduction and abduction functions of the MCP joint.

Through the aforementioned principles of multi-component coordination and motion control, the design requirements for the index finger mechanism’s three degrees of freedom (DOFs) can be met.

### 2.1. Specific Implementation of Design Structures

To achieve the design objectives of a single-finger structure, four key elements must be coordinated at the system level: core component selection and parameter matching, efficient design of the power transmission path, rational planning of structural layout space, and adaptation of manufacturing processes and maintenance convenience. Based on these elements, developing a single-finger structure with stable motion characteristics and high robustness is the core prerequisite for ensuring its operational performance.

**Figure 3:**
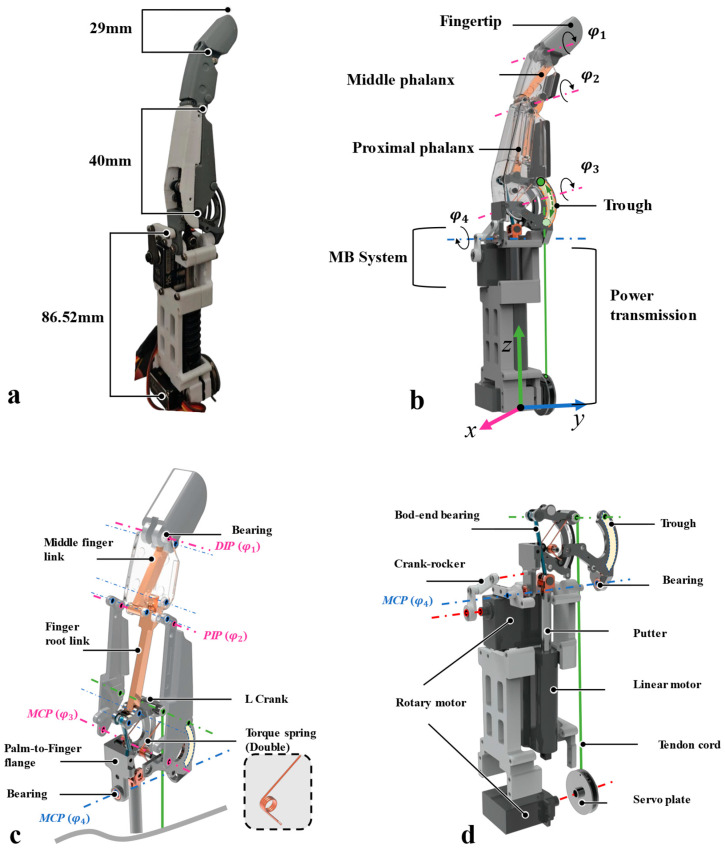
Detailed implementation of index finger unit design: outline of its structural system, with exploded views supporting machining and assembly. (**a**) Physical dimension drawing of the index finger unit, offering precise machining references; (**b**) modeling schematic, visually showing the overall structural layout; (**c**) exploded view of the three finger joints, clarifying component composition and assembly relationships; (**d**) exploded view of the integrated drive unit, guiding drive module assembly.

Figure 3 shows the index finger’s integrated design: it has a humanoid three-joint structure, with the power transmission mechanism modularly integrated in the palm. Physical isolation between joints and the power system simplifies component machining, reduces the impact of manufacturing tolerances on performance, and eases maintenance—allowing quick replacement of individual parts without full disassembly. Figure 3b presents the finger joint’s exploded view: The main joint body and connecting rod form a kinematic pair directly, building the basic power transmission linkage. The L-shaped link acts as both power transmitter and pivot support, enabling coordinated control of the connecting rod and tendon-cord drive. Two symmetric torsion springs solve the tendon-cord drive’s reset issue.

Figure 3c shows the exploded view of the single-finger power transmission system. It adopts an interleaved layout of two rotary motors (for lateral swing and tendon-rope drive) and one linear motor (for linkage structure drive). Optimized spatial configuration maximizes palm space utilization and avoids motor motion interference.

For key component functionality, the ball-head link is the power transmission core: its rotating pair enables multi-range vertical-plane motion, and its ball-head joint has −15 to 15° multi-directional offset to adapt to MCP joint (metacarpophalangeal joint) lateral movement without interfering with the PIP and DIP joints. Additionally, the L-shaped crank’s right node forms a sliding pair with the metacarpal groove; the groove’s travel matches the MCP joint’s 0–90° motion envelope, ensuring precise articulation and reliable position limits.

The index finger’s heavy operational load requires a high actuator output force. High-strength metal rods and shafts act as core load-bearing components, meeting strength needs with a compact, simplified structure to avoid excessive space use. Its multi-DOF design exposes joint connections to combined torque, bending moment, and axial loads, whose strength affects overall service life and safety. To balance joint load capacity and size constraints, precision bushings form the core joint connection—their tight fit with shafts enhances wear resistance and impact tolerance, while minimizing clearance reduces motion errors for smooth operation

### 2.2. MCP Joint Structure

In the index finger’s 3DOF structure design, simple machinable rods enable DIP-PIP joint coupling drives to balance manufacturing ease and cost. The MCP joint’s lateral swing and bending reset rely on two core structures: a torsion spring (tendon-rope reset, with reliable restoring force) and a crank–rocker mechanism (MCP adduction/abduction, defining movement flexibility and range). As these two structures are indispensable for the single finger’s 3DOF motion, their systematic analysis and design are essential to ensure the system’s motion performance.

#### 2.2.1. Torsion Spring Design

During metacarpophalangeal joint flexion/extension, tendon-cord contraction pulls the L-shaped linkage to bend the proximal interphalangeal joint and compresses the torsion spring for energy storage. When the tendon-cord relaxes, the torsion spring releases potential energy to generate counter torque, driving the L-shaped linkage in reverse and finally resetting the joint from flexion to extension. Thus, the torsion spring is the core component providing “reset power” for the mechanism [19,20].

**Figure 4:**
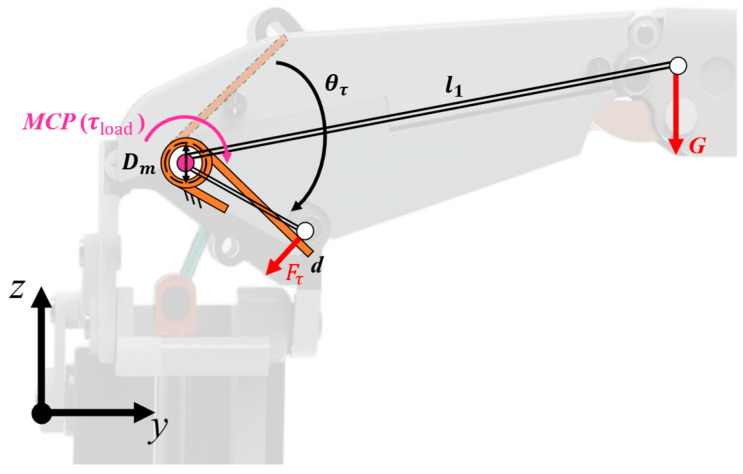
Schematic diagram of the gravitational force acting on the finger joint.

Analysis shows that the torsion spring reset must counteract the torque from finger weight at the MCP joint. Maximum torque occurs when the MCP joint is flexed at 90° and both interphalangeal joints are extended, with the gravity principle at this position shown in Figure 4. Total finger weight and center of gravity are obtained via the SolidWorks-2021 mass evaluation module. The lever arm formed by the center of mass and the MCP joint is $l_{1}=59.14\mathrm{mm}$. The gravitational force acting on the center of mass is G=0.426N. Therefore, the moment generated at the MCP joint is $\tau_{\mathrm{load}}=25.19N\cdot \mathrm{mm}$

(1) $$\tau_{\mathrm{load}}=l_{1}G$$

To ensure efficient, reliable torsion spring reset power output and avoid reset lag/failure, design conventions and standards typically require 1.5× working torque ($\tau_{\mathrm{load}}$) as pre-reserve torque at reset initiation (MCP joint fully bent, spring maximally deformed); reserve torque must meet $\tau_{pre.}\geq 1.5\tau_{\mathrm{load}}$. This design redundancy compensates for mechanical friction, assembly tolerances, and gravitational torque fluctuations to ensure smooth reset.

Based on the torque calculation formula for torsion springs, with the fundamental variable, and incorporating the $\tau_{pre.}\geq 1.5\tau_{\mathrm{load}}$ range obtained from the aforementioned calculations, the key design parameters for the torsion spring were ultimately determined through parameter iteration and performance verification. The specific parameters are shown in Table 3.

(2) $$k=\frac{Ed^{4}}{3660\;n\;D_{m}}$$

(3) $$\tau_{pre.}=k\theta_{\tau }$$

#### 2.2.2. Crank–Rocker Design

**Figure 5:**
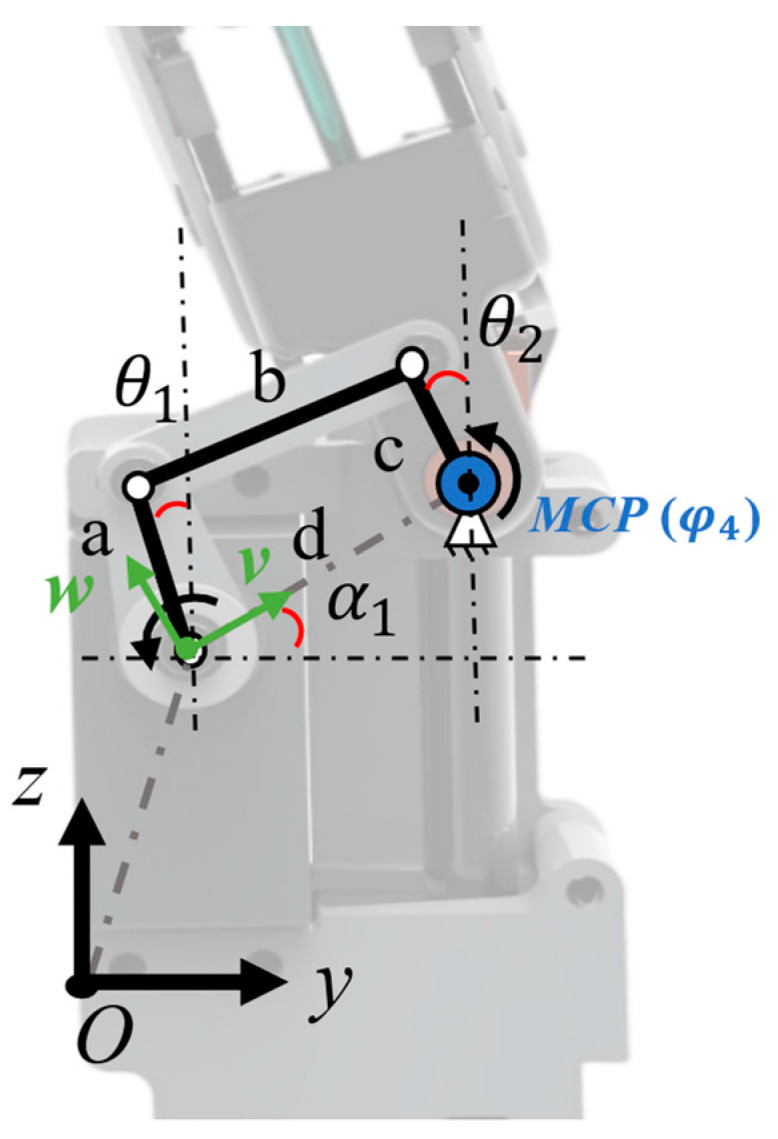
Principle analysis of the side-swing mechanism. The abduction and adduction movements of the MCP joint are achieved through a crank–rocker mechanism.

In designing the MCP joint (metacarpophalangeal joint) adduction–extension side-swing crank–rocker mechanism, the core motion path is as follows: the crank swings periodically to drive the rocker via motion coupling, achieving the desired MCP joint movement (mechanism principle in Figure 5). The mechanism comprises four key components—driving link (a), connecting rod (b), driven link (c), frame (d)—interconnected by rotary joints to form a closed kinematic chain.

Considering motion space constraints (avoiding interference, meeting MCP joint range) during mechanism operation, a local coordinate system is established with frame d as reference (α = 30° with world coordinate system). Component stroke ranges are in Table 4; in dimensional design, frame d (17 mm) and rocker c (7.6 mm) lengths are known inputs. Crank and connecting rod b lengths are critical to determine, as their dimensions directly dictate MCP joint lateral swing.

This design uses the Freudenstein linear equation (Equation (4)). Based on the planar four-bar mechanism’s kinematic relationships, it establishes a quantitative correlation between component length parameters and angular displacement. Three sampling points (Table 4) are substituted to form a 3 × 1 linear overdetermined system, and the least-squares solutions for *k*_1_ and *k*_3_ are obtained (Equation (8)). Where A is a 3 × 2 matrix $\left[\cos\theta_{1,i}\;1\right]$, and B is a 3 × 1 matrix $\left[\cos(\theta_{1,i}-\theta_{2,i})\cdot k_{2}\right]$.

(4) $$k_{1}\cos\theta_{2,i}-k_{1}\cos\theta_{2,i}+k_{3}=\cos(\theta_{1,i}-\theta_{2,i})$$

(5) $$k_{1}=d/a$$

(6) $$k_{2}=d/c$$

(7) $$k_{3}=(a^{2}-b^{2}+c^{2}+d^{2})/(2ac)$$

(8) $$A\cdot {\left[k_{1},k_{3}\right]}^{T}=B$$

Using the solved coefficients k_1_ and k_3_ and the coefficient-component length mapping from the Freudenstein equation derivation, inverse calculation gives crank *a* = 15 mm and connecting rod *b* = 9.6 mm, finalizing the mechanism’s core parameters.

### 2.3. Joint Angle Limitations

To satisfy the precise positioning demands of dexterous hand joints, this design adopts a mechanical limiting solution. Via targeted structural optimization, controllable motion range constraints for each joint are realized. The limiting mechanisms for specific joints are detailed below:

#### 2.3.1. Flexion Angle Limitation Design for DIP and PIP Joints

**Figure 6:**
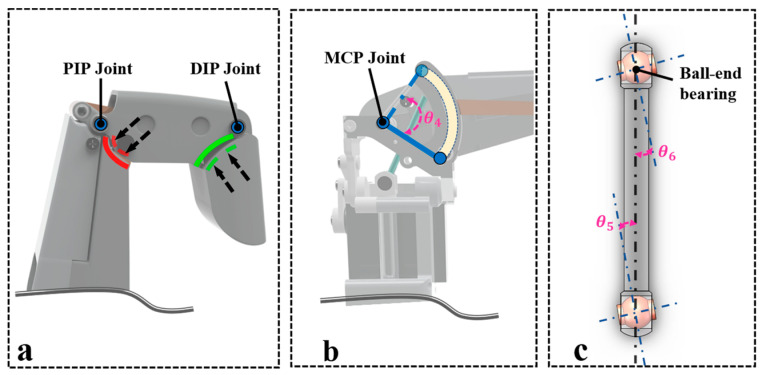
This illustrates joint motion limiters for safe articulation: Panel (**a**) depicts DIP/PIP joint limiters; Panel (**b**) shows MCP joint flexion/extension constraints, and Panel (**c**) illustrates the ball-and-socket linkage’s lateral swing limiting mechanism.

DIP and PIP joint bending angles are limited by an integrated shell structure. As shown in Figure 6a, the shell incorporates limiting grooves matching the joints’ motion trajectory, with same-color edges marking the mating surfaces of moving components and grooves. During flexion, the moving component rotates until contacting the groove’s end face; mechanical contact prevents over-flexion, precisely confining the bending angle to the designed threshold for safe, controllable motion.

#### 2.3.2. Bidirectional Motion Limitation Design for MCP Joint

As the core motion joint of the dexterous hand, the metacarpophalangeal (MCP) joint requires independent limitation for both flexion/extension and lateral swing motions. Therefore, a categorized limitation design approach is adopted, detailed as follows:

(1) Slotted Guide for Flexion–Extension Movement

The MCP joint’s flexion–extension uses a slotted mechanism: its base features an arc-shaped groove matching the movement trajectory, in a sliding fit with the joint axis (Figure 6b). During flexion/extension, the axis slides along the groove, with its end faces acting as limits to mechanically restrict travel. This precisely confines the flexion–extension angle to 0–90°, meeting gripping range requirements while preventing structural damage from over-flexion/extension.

(2) Spherical-Pivot Rod-Type Lateral-Swing-Limiting Mechanism

The MCP joint’s lateral swing, driven by the aforementioned crank–rocker mechanism, is angularly limited via a ball-rod assembly (Figure 6c). Spherical joints at both ends of the rod form an equivalent universal joint to adapt to spatial angular changes during the swing, with the rod connecting the proximal joint assembly and eccentric drive. Defining a and b as bidirectional deflection angles, the composite lateral swing angle is precisely confined to −15° ≤ a + b ≤ 15° through the ball-rod’s length constraints and the eccentric structure’s stroke limits.

In summary, this design achieves angle limitation for the DIP/PIP joints and dual-direction limitation for flexion/extension and lateral swing of the MCP joint through mechanical structure optimization tailored to the distinct motion characteristics of each joint.

## 3. Simulation Analysis of Finger Units

### 3.1. Drive Performance Verification

Part One completed the conceptual design and physical implementation of the index finger’s 3DOF hybrid-drive structure. However, fingertip force and gripping force are core end-effector performance indicators for dexterous hand operation, and drive motor selection directly determines finger performance. Thus, mechanical performance verification of the single finger’s drive motors is necessary.

Due to the index finger’s “cable drive + linkage” hybrid mechanism, plus the rod-coupled system’s long transmission path and complex structure, static mechanical estimation cannot accurately reflect motor dynamic forces. Thus, this study builds a “fingertip load force–driver output force” dynamic model and simulation system: apply a constant 20 N vertical load to the fingertip, monitor driver real-time output force during the finger’s 3-DOF full-range motion; for grasping force, simulate knuckle contact surface load distribution to replicate grasping-force patterns and validate motor grasping performance. Additionally, separate validation of lateral fingertip load is needed to fully verify motor mechanical reliability, with subsequent sections explaining these procedures in detail.

#### 3.1.1. Linear Drive Motor Calibration

**Figure 7:**
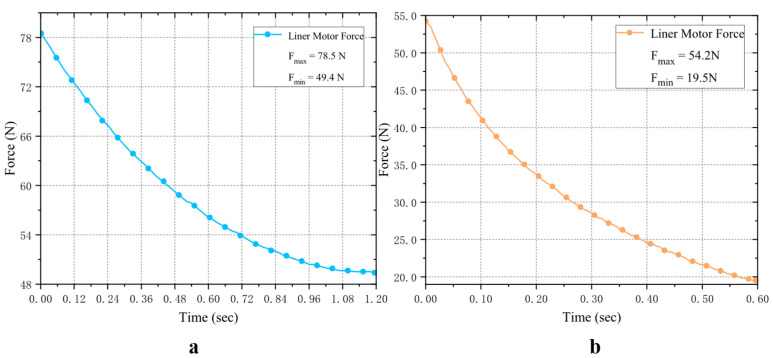
Linear motor output force monitoring. (**a**) shows the motor output value when the MCP joint is extended; (**b**) shows the motor output value when the MCP joint is fully flexed. Where $F_{l}\geq 1.2F_{\max}$, and with the MCP joint fully flexed, the required driving force is $F_{l}\geq 1.5F_{\max}$. Therefore, this linear motor meets the requirements for a single-finger unit.

First, for the DIP-PIP joint coupled-motion linkage system, a rated-thrust electric push rod is used as the linear motor. In dynamic simulation (Figure 7), Figure 7a shows the application of a max-20 N fingertip load [9,21,22] under MCP joint extension and full flexion, achieving joint coupling; Figure 7b. shows the linear motor’s output force under both conditions. More output force is needed during MCP extension, so this state’s max driving force is used for verification, with a 20% motor margin per dexterous hand requirement.

#### 3.1.2. Rotary Drive Motor Calibration

**Figure 8:**
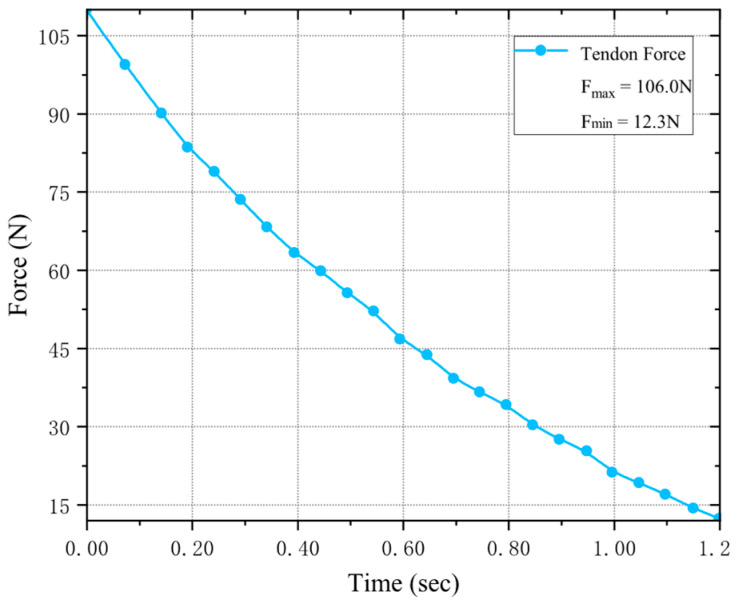
Rotating motor output force monitoring, driver output force values at DIP joint extension state.

In the index finger unit, both the tendon-cord drive (MCP flexion) and lateral swing drive (MCP adduction/abduction) require one rotary motor each. The selected motors have a rated torque of 14.0 kgf·cm and a no-load speed of 0.09 s/60°. Refer to Figure 8 for the tendon-cord drive motor calibration. To maintain stable and reasonable fingertip force output during operation, the MCP joint must not relax under applied load. Therefore, we maintain a fingertip load of F = 20 N. Since the PIP and DIP joints exhibit maximum lever arms and the highest tendon-cord force requirements during extension, we simulate this condition to monitor the required tendon-cord tension across the MCP joint’s full flexion range from initial to fully bent positions.

We can directly determine the maximum driving force of the rope as F = 106 N. The radius of the force arm for our pulley (*r*) is 10 mm. As shown in Equation (9), the motor’s driving force meets our requirements with a 20% margin.

(9) $$1.2F_{need}\leq T_{R}\cdot r$$

For the crank–rocker mechanism of the pendulum tester, based on scholarly research [23,24], we know that human fingers exert lateral forces during operation, with a maximum load of (1.5–12.6 N). Using the principle of virtual work, we can estimate the required torque ($T_{in}$) of the crank through Equation (10).

(10) $$T_{in}=T_{out}\cdot \frac{\omega_{c}}{\omega_{a}}$$

#### 3.1.3. Grip Pattern Verification

**Figure 9:**
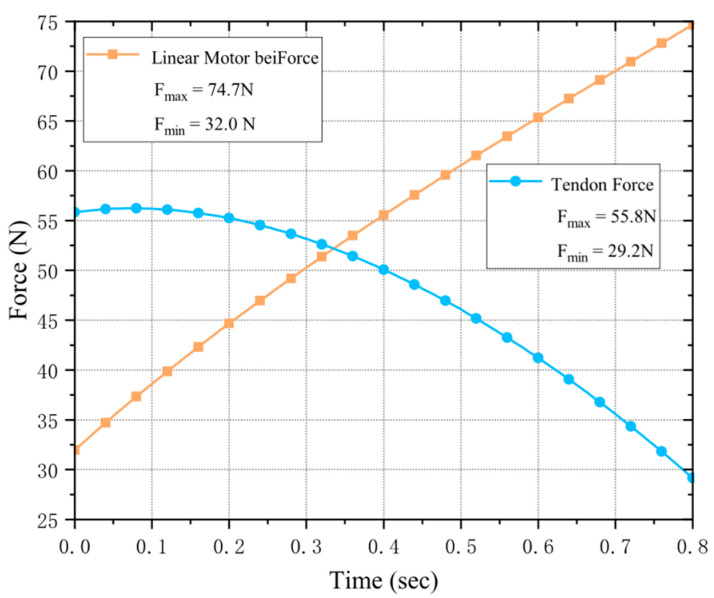
Output force monitoring of linear motor and rotary motor during grasping process.

Another critical validation involved simulating the finger’s grasping capability. Research indicates that the maximum overall operating force of the finger occurs at the midpoint of each joint, specifically at a rotation angle of 45° [18,23,25]. Therefore, we simulated the finger’s movement from this position to the end position and validated its grasping capability, as shown in Figure 9.

Apply a 20 N load to each fingertip to obtain the moment-force output of the link-driven actuator and tendon-driven actuator. By collecting data, ensure that the required output force for heavy-load gripping is significantly less than the actuator’s rated value.

### 3.2. Transient Analysis of Key Structures

The aforementioned verification indicates that the selected motor’s output capacity meets design requirements. However, motor performance compliance does not guarantee operational safety of the finger structure. Therefore, further structural safety analysis of the hybrid-driven single finger is necessary. This study focuses on the linkage system not because it bears the highest load, but because it serves as the “sole rigid force transmission bridge” within the hybrid-drive architecture.

**Figure 10:**
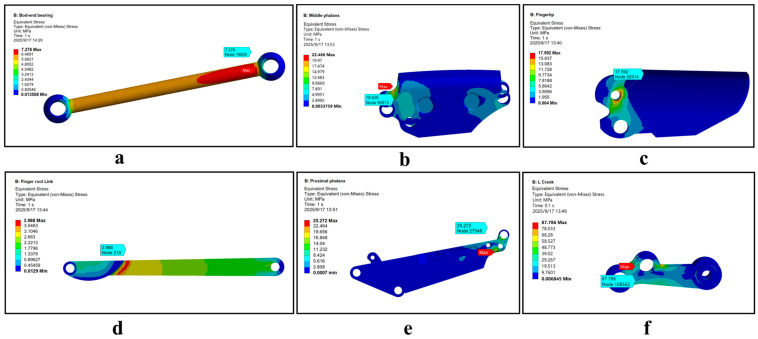
Transient simulation of the joint structure of the index finger’s third phalanx. (**a**) Force simulation of the tie rod; (**b**) Structural simulation of the middle phalanx; (**c**) Structural simulation of the interphalangeal joint; (**d**) Structural simulation of the linkage mechanism; (**e**) Structural analysis of the metacarpal region; (**f**) Force analysis of the L-shaped linkage.

Specifically, the DIP-PIP joint coupling precision and stable fingertip force output both rely on the linkage system’s transmission performance and structural integrity. Insufficient strength or excessive deformation of critical components (ball-and-socket linkages, L-shaped cranks) or core connections (four-bar hinges) will degrade single-finger performance or cause failure, increasing maintenance costs and troubleshooting complexity while undermining long-term stability. Thus, this study uses ANSYS (2020 version) Workbench to perform transient dynamic analysis on these critical components and hinges, ensuring they meet design safety requirements (Figure 10).

We set the link drive and tendon drive forces to their maximum values, defined the transient simulation step size, and monitored stress–strain during motion. We focused on stress concentration and high-deformation-risk areas in critical components to assess safety; critical components utilize TC4 commercial titanium alloy (ultimate fatigue strength 420–460 MPa). Calculations confirm its safety factor meets structural reliability requirements S≥4.784. Consequently, the linkage system driving the DIP and PIP joints exhibits sound design safety, capable of satisfying the long-term stable operation demands of the joint drive system.

## 4. Kinematics Analysis of the Index Finger Unit

**Figure 11:**
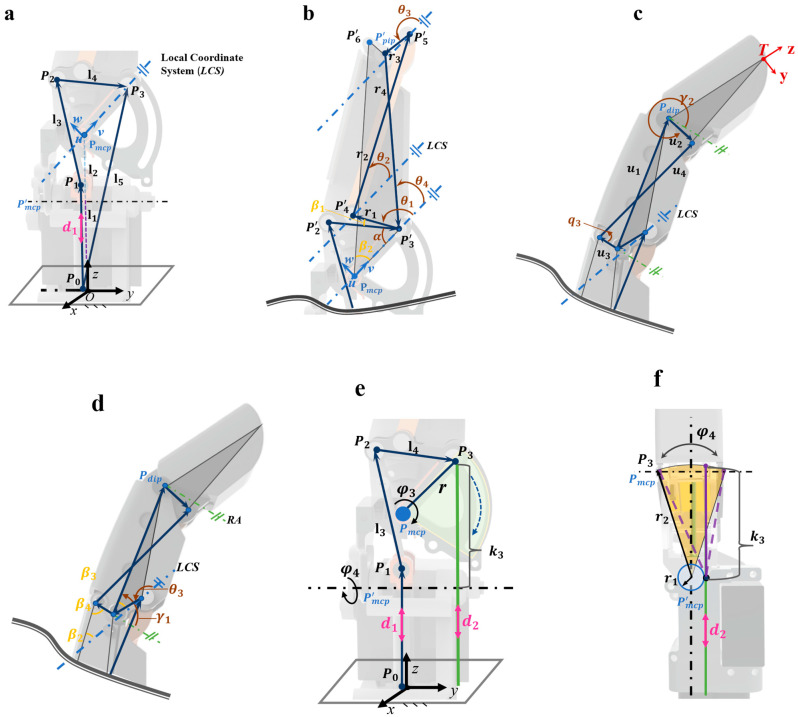
Schematic diagram of kinematic analysis for each joint. (**a**) Schematic diagram of metacarpophalangeal (MCP) joint kinematics; (**b**) Schematic diagram of proximal interphalangeal (PIP) joint kinematics; (**c**,**d**) Four-bar linkage connecting the proximal and distal interphalangeal joints; (**e**,**f**) Metacarpophalangeal joint for flexion/extension and lateral swing motion.

To theoretically analyze the proposed mechanism, we divide it into four motion models: the MCP joint, as shown in Figure 11a, the PIP joint, as depicted in Figure 11b, a four-bar linkage coupling the PIP and DIP joints (Figure 11c,d), and the MCP flexion/extension and lateral-swing joints as illustrated in Figure 11e,f. Based on these four models, inverse kinematic analysis of the mechanism is conducted, which is crucial for validating the rationality of the mechanism design and achieving precise motion control.

For the metacarpophalangeal joint (MCP) at the base of the finger, it must achieve two degrees of freedom: flexion/extension and lateral swing. When considering only flexion/extension without lateral swing, the drive stroke of the rope-driven system is $K_{t}$, expressed as shown in the equation, where $K_{3}$ is the initial distance, as shown in Figure 11d.

(11) $$k_{t}{=k}_{3}-r[\sin\frac{\pi }{4}-\sin(\frac{\pi }{4}-\varphi_{3})]$$

However, during actual index finger movement, the MCP joint often has lateral-swing freedom (Figure 11e). Due to the eccentric arrangement of the tendon-rope relative to the central axis by r1, the rope drive’s actual stroke must account for length adjustments from lateral swing: when the MCP joint swings right, structural geometry changes shorten the rope drive path ($d_{2}$), causing stroke redundancy. The length of the route change can be equivalently regarded as the section marked purple b in Figure 11f. The corrected expression is as follows:

(12) $$d_{2}=\frac{r\varphi_{3}\pi }{180}-D_{1}$$

(13) $$D_{1}=k_{t}-\sqrt{{\left[r_{2}\sin(\varphi_{4})-r_{1}\right]}^{2}+{\left[r_{2}\cos(\varphi_{4})\right]}^{2}}$$

When the MCP joint swings left, the lateral swing bearing’s mechanical limit elongates the rope drive path, requiring an extra drive stroke to ensure transmission effectiveness. Thus, its full stroke expression is in Equations (14) and (15), where $d_{2}$ is the rope drive’s required adjustment length.

(14) $$d_{2}=\frac{r\varphi_{3}\pi }{180}+D_{2}$$

(15) $$D_{2}=[\sqrt{r_{2}^{2}-r_{1}^{2}}+\frac{[\frac{\pi }{2}-(\arctan\frac{\sqrt{r_{2}^{2}-r_{1}^{2}}}{r_{1}})+\left|\varphi_{4}\right|]\pi r}{180}-k_{t}]$$

The distal interphalangeal joint (DIP) and proximal interphalangeal joint (PIP) achieve coupled motion through an integrated four-bar linkage mechanism, as shown in Figure 11b,c. Power and motion are transmitted via an L-shaped link from a linear motor. In actual motion, the lateral sway of the index finger has no significant effect on the geometric configuration of the linkage system. Therefore, the inverse kinematics solution for the DIP and PIP joints requires joint analytical processing.

To clarify the kinematic characteristics of the DIP joint, its kinematic calculation structure is decomposed into the simplified model in Figure 11c,d, where $u_{1}\sim u_{3}$ represent the vector parameters of the four-bar linkage. A reference axis RA (Figure 11c) is introduced to precisely monitor DIP joint angular variation. Color coding distinguishes angular attributes: variable angles (brown) and fixed angles (yellow), clarifying dynamic–static parameter boundaries.

**Figure 12:**
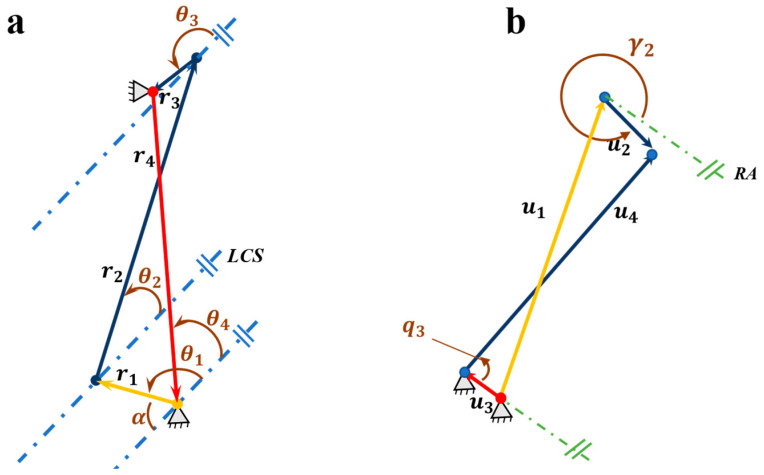
Four-bar linkage extraction diagram for driving the PIP and DIP joints. (**a**) Schematic diagram of the four-bar linkage mechanism driving the middle phalanx movement; (**b**) Schematic diagram of the four-bar linkage mechanism driving the fingertip movement.

Based on this model, vector closure equations (Equation (16)) are established. Decomposing these into horizontal and vertical components, solving, and reorganizing completes kinematic analysis—essentially equivalent to the direct kinematic solution of a mechanical four-bar linkage. The core four-bar driving mechanism (Figure 12b) is extracted: red $u_{3}$ is the fixed frame, yellow $u_{1}$ the active element.

This solution provides critical input for subsequent inverse kinematics of adjacent joints. From it, analytical expressions for DIP joint angular displacement are derived. Incorporating geometric constraints (Figure 11b), analytical relationships between $\theta_{3}$ and other motion angles are established.

(16) $$u_{1}+u_{2}=u_{3}+u_{3}$$

(17) $$\theta_{3}=\gamma_{1}+\beta_{2}-\beta_{3}+\beta_{4}$$

Among them,

(18) $$\gamma_{1}=\arcsin\left(\frac{-u_{2}S\gamma_{2}+u_{4}Sq_{3}}{u_{1}}\right)$$

(19) $$\gamma_{2}=\arcsin\left(\frac{u_{4}^{2}+u_{2}^{2}+u_{3}^{2}-u_{1}^{2}-2u_{3}u_{3}Cq_{3}}{\sqrt{{\left(2u_{2}u_{4}Sq_{3}\right)}^{2}+{\left(2u_{2}u_{4}Cq_{3}-2u_{2}u_{3}\right)}^{2}}}\right)-\arctan\frac{2u_{2}u_{4}Cq_{3}-2u_{2}u_{3}}{2u_{2}u_{4}Sq_{3}}$$

For the proximal interphalangeal joint (PIP), similarly based on the principle of vector sum and equality in link mechanisms, its vector closure equation is constructed, with separate component equations listed for the horizontal and vertical directions. The four-bar linkage diagram driving the PIP joint is shown in Figure 12a. Similarly, $r_{4}$ represents the frame, and $r_{1}$ represents the driven end. By solving the simultaneous equations, the analytical expression for the angular displacement of the PIP joint is obtained, as shown in Equation (20).

(20) $$r_{1}+r_{2}=r_{3}+r_{4}$$

(21) $$\theta_{1}=\arcsin\left(\frac{r_{2}^{2}-r_{1}^{2}-r_{3}^{2}-r_{4}^{2}+2r_{3}r_{4}C\theta_{3-4}}{\sqrt{{\left[2r_{1}(r_{3}S\theta_{3}-r_{4}S\theta_{4})\right]}^{2}+{\left[2r_{1}(r_{3}C\theta_{3}-r_{4}C\theta_{4})\right]}^{2}}}\right)-\arctan\left(\frac{2r_{1}(r_{3}C\theta_{3}-r_{4}C\theta_{4})}{2r_{1}(r_{3}S\theta_{3}-r_{4}S\theta_{4})}\right)$$

Furthermore, in Figure 11a we construct the vector relationship $P_{01}+P_{12}+P_{23}=P_{03}$. Vector $P_{23}$ is obtained by transforming the local coordinate system u−v into a global coordinate system x−y. The detailed expression of vector $P_{23}$ is shown in Equation (22).

(22) $$P_{23}=RP_{23}^{\prime }=\left[\begin{array}{c} l_{4}Sq_{2}S(q_{1}-\alpha )\\ l_{4}C(q_{1}-\alpha )\\ l_{4}Cq_{2}S(q_{1}-\alpha ) \end{array}\right]$$

(23) $$\begin{array}{cc} P_{03} & =P_{3}-P_{0}\\ & =(RP_{23}^{\prime }+P_{mcp})-P_{0}\\ & =\left[\begin{array}{c} b_{y}\sin q_{1}\sin q_{2}+l_{2}Cq_{2}\\ b_{y}\cos q_{1}-c_{q}\\ b_{y}\cos q_{2}\sin q_{1}+l_{2}Sq_{2}+l_{1} \end{array}\right] \end{array}$$

Since we know $\left\|P_{12}\right\|=l_{3}$, we obtain the equation $P_{12,x}^{2}+P_{12,y}^{2}+{\left(P_{12,z}-d_{3}\right)}^{2}=l_{3}^{2}$. After simplifying and calculating, we derive the motion equation for the linear motor as shown in Equation (24). This equation maps the motion requirements of the terminal phalanx to the input commands of the drive unit, providing a theoretical basis for the design of the control system.

(24) $$d_{3}=P_{12,z}\pm \sqrt{l_{3}^{2}-P_{12,x}^{2}-P_{12,y}^{2}}$$

**Figure 13:**
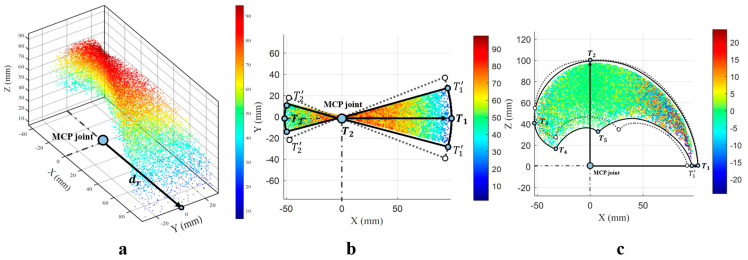
Schematic diagram of the movement space: (**a**) is the 3D view, (**b**) is the top view, (**c**) is the side view.

To validate the working space of the designed robotic finger, an analysis of its reachable working space is conducted, as shown in Figure 13a–c. Figure 13a displays a three-dimensional view of the reachable working space composed of points attainable by the fingertip. Here, dr represents the distance from the fingertip to the fixed MCP joint of the finger, which is taken as the origin. Using the same origin, we adjusted the lever arm length to match the dimensions of a human index finger and compared its motion space with that of our designed dexterous-hand index finger, as shown in Figure 13b,c. The dashed lines indicate the motion space of the human index finger. Figure 13c presents a side view of the robotic finger’s motion space, while Figure 13b shows a top view of the robotic finger’s motion space.

## 5. Experimental Testing

Motor function and actuation force capability are the core dimensions defining the performance of the dexterous hand’s index finger unit, directly determining its operational capacity and influencing the overall hand’s functionality. Previous research has completed structural design, dynamic, and kinematic analyses of the index finger, providing theoretical characterization support for joint performance. However, practical factors such as structural machining errors and friction wear in transmission components may cause discrepancies between theoretical and actual performance, necessitating an evaluation of the index finger joint’s real-world functionality. Thus, this section first fabricates the dexterous hand’s index finger monofinger structure, then experimentally evaluates key performance metrics including degrees of freedom, joint motion verification, active/passive force testing, and fingertip force measurement. These results provide a reliable foundation for subsequent index finger joint structure optimization, control strategy refinement, and enhancement of the dexterous hand’s overall operational performance.

### 5.1. Physical Function Test

Kinetic performance is a core factor in evaluating the dexterity of the index finger’s operational flexibility. It requires experimental verification of the range of motion at each joint and multi-degree-of-freedom capabilities to establish the kinematic foundation for subsequent operational force and fingertip force testing. However, practical factors such as structural manufacturing errors and friction losses in transmission components may cause discrepancies between theoretical and physical performance. Therefore, the actual performance of the index finger joints must be assessed.

**Figure 14:**
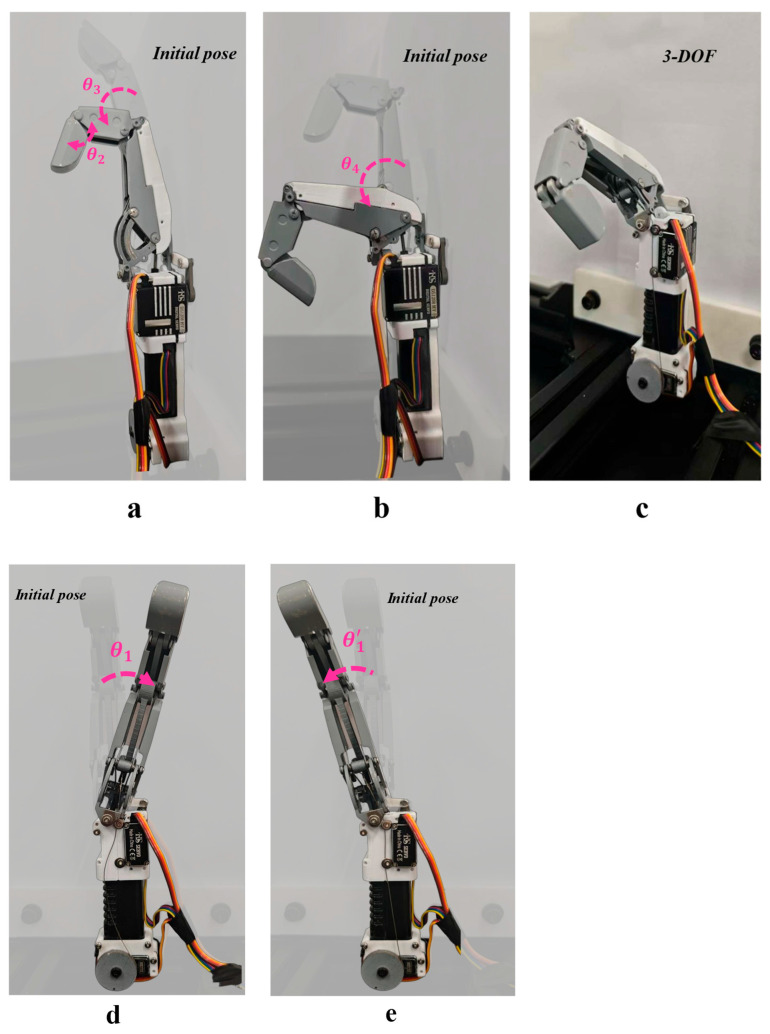
Joint mobility tests, joint range of motion testing, to verify the mobility of each index finger joint. (**a**) Movement testing of the DIP and PIP joints; (**b**) represents the flexion and extension movement of the MCP joint; (**c**) represents a 3-degree-of-freedom display; (**d**,**e**) represent lateral-swinging motion of the fingers.

For the proximal interphalangeal (PIP) and distal interphalangeal (DIP) joints, flexion-coupled underactuated testing was employed to measure their bending angles. Figure 14a illustrates the motion correlation between the DIP and PIP joints under underactuated transmission. The maximum bending angle of the DIP joint ($\theta_{2}$) is 80°, while the maximum motion angle of the PIP joint ($\theta_{3}$) is 90°. This angular range satisfies the envelope requirements for the fingertip when grasping objects with dexterity [26]. Extending the range of motion of the metacarpophalangeal (MCP) joint is crucial for expanding the index finger’s longitudinal operational capabilities. As shown in Figure 14b, the MCP joint’s flexion/extension was tested via the drive unit, achieving a measured flexion/extension angle ($\theta_{4}$) of 83°—providing ample motion redundancy for the index finger to grasp objects at varying heights.

The lateral swing motion of the MCP joint enhances the index finger’s adaptability in lateral spatial orientation. Figure 14d,e. illustrate the lateral swing states during abduction and adduction of the MCP joint, respectively. This motion capability enables the index finger to adjust its relative position with other fingers or manipulated objects in the lateral dimension, thereby improving collaborative precision. Figure 14c presents an overall schematic of the 3-DOF single-finger structure for the index finger. Combined with joint motion test results, this validates that the designed single-finger structure achieves the anticipated multi-degree-of-freedom motion performance, establishing the kinematic feasibility foundation for subsequent active/passive and fingertip force testing.

During simulated motion, the maximum bending angle of the MCP joint ranges from 85 to 90 degrees. In actual experiments, after maintaining 15 repetitions of MCP motion, the observed movement angle is clearly between 80 and 83 degrees. Analysis indicates this discrepancy primarily stems from rope friction losses during actual transmission and manufacturing/assembly tolerances, with the overall deviation falling within acceptable engineering limits. For the operating speed of the rope-driven MCP joint, a stopwatch was used to record the time required for the joint to complete a full cycle over five repeated measurements. The measured movement speed was 104°/s.

**Figure 15:**
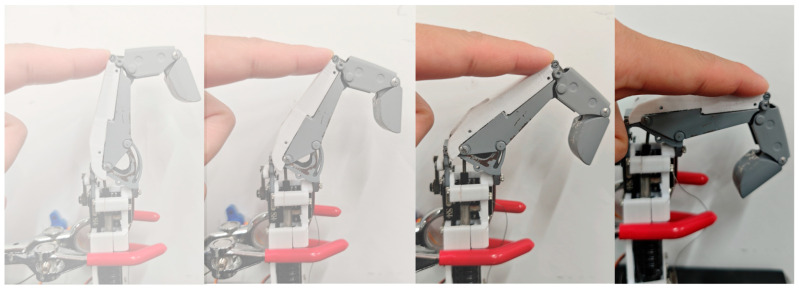
Joint impact resistance demonstration. Demonstrates passive bending capability under applied force.

Through the rational integration of a linkage mechanism and tendon-rope drive, this index finger structure possesses a certain degree of impact resistance. To validate this performance, experiments employed a human hand as the resistance application unit to exert external force on the knuckle unit. Under this load, the finger joint achieves adaptive bending. As shown in Figure 15, the experimental results demonstrate that this design meets the requirements for impact-resistant self-protection functions in industrial environments.

### 5.2. Operational Force Testing

Operational force performance serves as the core metric for the dexterous hand’s index finger to accomplish tasks such as grasping and load handling. It encompasses two key performance categories: active grip force and fingertip force, validated through the following experiments.

**Figure 16:**
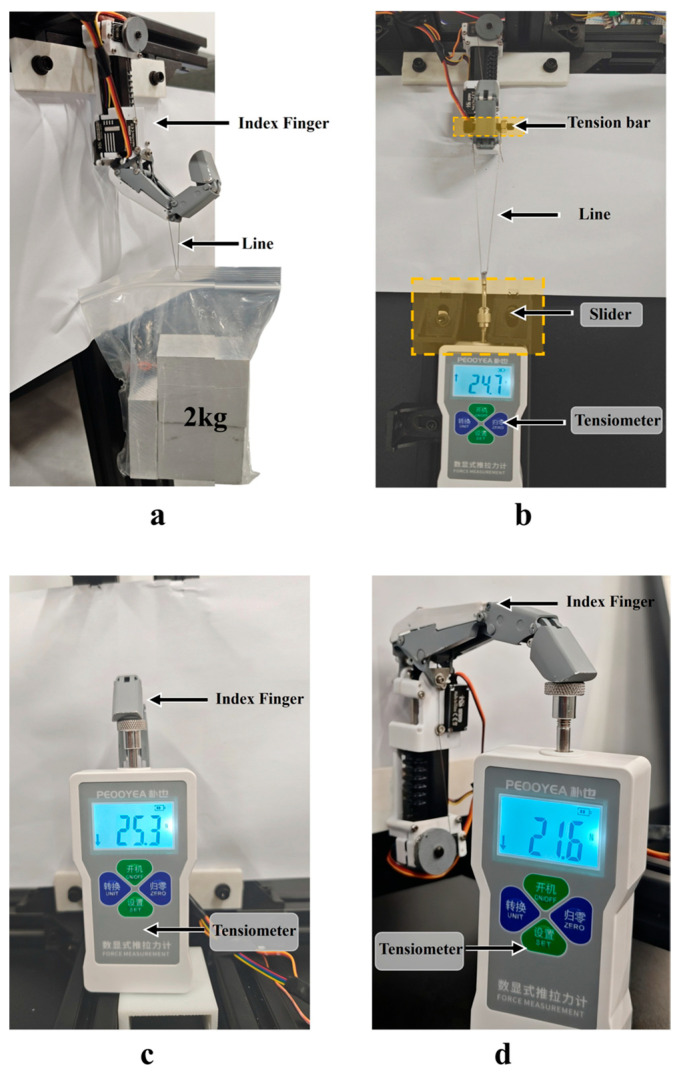
Index finger unit operational force experiments: (**a**) Passive force testing: verifying structural stiffness and resistance; (**b**) Active force testing: validating drive system output capability; (**c**) Tip force testing (MCP extended): evaluating extended-posture load performance; (**d**) Tip force testing (MCP flexed): assessing flexed-configuration load output.

For the passive grip strength test, to simulate the operational scenario of a human index finger lifting heavy objects, weight bags of 1.2 kg, 1.6 kg, and 2 kg were suspended from the fingertip using fishing line, as shown in Figure 16a. The servo system was activated to enable the index finger to complete a 20 s stable grasping action. No functional issues such as structural loosening or transmission failure occurred, providing preliminary validation of its performance.

Active grip strength testing is conducted using the active grasping test platform to measure pulling force (Figure 16b): First, secure the tip of the index finger to the upper end of an aluminum profile, with a digital force gauge positioned at the lower end. Use the profile’s sliding block to limit the force gauge’s shoulder. The force gauge’s output end is connected to a rod via fishing line, allowing the rod to rest naturally on the index finger’s surface. Subsequently, the index finger is flexed to grasp the pull rod while simultaneously adjusting the slider to retract the force gauge until the connecting rod displaces or can no longer effectively engage the pull rod. During connecting rod displacement, the force gauge displayed a maximum of 24.7 N, meeting the 20 N design specification. This validates the force transmission efficiency and structural reliability under index finger pulling conditions.

To verify the short-cycle stability of the structure, 15 consecutive grasp–release-cycle tests were conducted under the same experimental setup and environmental conditions. Each cycle included a complete process of finger flexion-grasping and extension-release, with the maximum pulling force recorded for each cycle. The test results showed that the maximum pulling force of the 15 cycles ranged from 23.9 N to 24.8 N, with a coefficient of variation (CV) of 1.2%, indicating no significant attenuation in force transmission efficiency.

To evaluate the force output capacity of the index finger, fingertip force tests were performed under flexion and extension postures of its metacarpophalangeal (MCP) joint. In flexion, a digital push–pull dynamometer was vertically loaded on the flexed fingertip until near structural failure, yielding a maximum force of 25.3 N (Figure 16c); the counterpart maximum force in extension reached 21.6 N (Figure 16d) under the same test protocol. For short-cycle stability verification, 15 consecutive load–unload cycles (loading to near-failure force, followed by full unloading) were implemented for each posture. Test results showed that the maximum forces ranged from 23.8 N to 25.9 N (coefficient of variation, CV = 2.2%) in MCP flexion, and from 20.2 N to 22.3 N (CV = 2.4%) in extension. Fluctuations were primarily attributed to minor tendon–cable friction and hinge contact resistance variations during cyclic motion, which aligns with the inherent characteristics of the hybrid-drive system. No structural loosening, joint jamming, or transmission wear was observed throughout the tests. These results confirm the reliable force output of the finger under different postures and its excellent short-cycle stability, which satisfies the requirements of repeated precision manipulation tasks.

The test results for fingertip force under two posture types indicate that the index finger maintains reliable fingertip force output across different operational postures. In summary, the active gripping force, pulling force, and fingertip force under various postures of the single-finger index finger structure all meet design expectations. This provides the force performance foundation for subsequent development of dexterous hands and execution of complex operational tasks.

## 6. Conclusions

This study focuses on the design of independent finger units within a dexterous hand. Taking the index finger unit as an example, a hybrid-driven 3-DOF index finger unit is proposed. In terms of joint motion performance, the MCP joint offers a 90° range of flexion/extension and ±15° of lateral rotation; the DIP and PIP joints ensure ≥80° of flexion/extension. Regarding operating force performance, it achieves ≥20 N of fingertip operating force and active/passive tensile force output. This design combines link-driven and tendon-driven mechanisms, effectively retaining the link-driven system’s capability to accommodate high operating forces while preserving the tendon-driven system’s flexible shock absorption. Additionally, the design of the pendulum structure ensures reliability for complex grasping tasks. Furthermore, the independent design of the index finger joint and drive unit offers advantages in manufacturing, maintenance, and iterative optimization. Experimental validation of its motion range, degree-of-freedom performance, and operational force capabilities confirms the structural design’s feasibility, laying the foundation for subsequent integration into a full five-finger dexterous hand. In design and implementation, the linkage mechanism is highly compatible with the tendon–cable drive. However, the eccentric cable layout requires integrating cable mechanical property calculations for lateral-swing motion, notably raising the drive’s motion control algorithm complexity and leaving room for optimization.

Existing single-finger designs meet basic needs but have two key limitations: incomplete tech transition from single-finger validation to whole-hand integration (blocking coordinated hand operation), and no multi-dimensional force sensors at fingertips/pads (hindering refined contact force data acquisition, limiting control algorithm optimization and complex-scenario precision). Subsequent research will focus on the “Whole-Hand Integration–Perception Fusion–Algorithm Optimization” framework: building on single-finger performance, it will advance whole-hand mechanical adaptation and collaboration, integrate force sensors for a comprehensive perception network, optimize algorithms via sensor data, and use cutting-edge research to solve bottlenecks—ultimately completing whole-hand design and validation to lay a tech foundation for practical applications.

## Figures and Tables

**Table 1:** Comparison of typical dexterous hands.

Feature	Proposed Design	ILDA Hand	Shadow Hand	Leap Hand	Inspire Hand	RoboNaut Hand
Driving type	LD	LD	TD	DM	LD	TD
Finger DOFs	3	3	4	4	1	4
Fingertip force(N)	25.3	34	10	5 N	30	21.85
Impact-resistant	O	X	O	X	X	O
Forearm	X	X	O	X	X	O
Finger length(mm)	98	101	105	85	81.3	127

Driving type: DM (Direct-motored), TD (tendon-driven), LD (linkage-driven); X denotes exclusion, while O denotes inclusion.

**Table 2:** Comparison of the human index finger and the index finger we designed.

	Finger Length (Index)				Finger Joint Range				Palm Size	
	$l_{p}$	$l_{m}$	$l_{d}$	Sum	MCP (abd/add)	MCP (flex/ext)	PIP	DIP	Width	length
	(mm)	(mm)	(mm)	(mm)	(deg)	(deg)	(deg)	(deg)	(mm)	(mm)
**Human**	48.3	28.2	19.1	95.6	±20	90	90	80	22.23	85.84
**Robot**	40	29	29	98	±15	83	90	80	22.40	86.52
**Ratio between human and robotic hands**										
	17.2%	2.8%	51.8%	2.5%					0.76%	0.79%

$l_{p}$: Proximal phalange length, $l_{m}$: middle phalange length, $l_{d}$: distal phalange length.; **MCP (abd/add)**: the abduction/adduction displacement angle of the MCP joint; **MCP (flex/ext)**: the flexion/extension displacement angle of the MCP joint.

**Table 3:** Parameters of torsion spring.

Joint	MCP
**Material**	SUP9-WPB
**E(N/mm2)**	200,000
**d(mm)**	0.6
**Number of laps n**	4
**Dm(mm)**	4.6
**Rotation angle(°)**	114
**Number of torsion springs s**	2
**Coefficient of elasticity k**	0.385
$\tau_{pre.}(N\cdot mm)$	87.78

**Table 4:** Crank–crank position sampling.

Sampling Point i	Crank Input Angle $\theta_{1}$	Joystick Output Angle $\theta_{2}$
1	30°	30°
2	20°	15°
3	40°	45°

## Data Availability

The original contributions presented in this study are included in the article. Further inquiries can be directed to the corresponding author.
